# Evaluation and Prediction of the Bending Behavior of Circular Hollow Steel Tube Sections Using Finite Element Analysis

**DOI:** 10.3390/ma15113919

**Published:** 2022-05-31

**Authors:** Manahel Shahath Khalaf, Amer M. Ibrahim, Hadee Mohammed Najm, Amer Hassan, Mohanad Muayad Sabri Sabri, Mohammed A. Alamir, Ibrahim M. Alarifi

**Affiliations:** 1 Department of Civil Engineering, College of Engineering, University of Diyala, Baqubah 32001, Diyala, Iraq; 2 Department of Civil Engineering, Zakir Husain Engineering College, Aligarh Muslim University, Aligarh 202002, India; gk4071@myamu.ac.in (H.M.N.); ameralburay@gmail.com (A.H.); 3 Peter the Great St. Petersburg Polytechnic University, 195251 St. Petersburg, Russia; mohanad.m.sabri@gmail.com; 4 Department of Mechanical Engineering, College of Engineering, Jazan University, Jazan 45142, Saudi Arabia; malamir@jazanu.edu.sa; 5 Department of Mechanical and Industrial Engineering, College of Engineering, Majmaah University, Al-Majmaah, Riyadh 11952, Saudi Arabia; i.alarifi@mu.edu.sa

**Keywords:** bending, local buckling, steel tubes, circular hollow section, finite element model

## Abstract

Circular hollow steel tube columns are widely used in high-rise buildings and bridges due to their ductility and lower weight compared to reinforced concrete. The use of this type of steel section has several advantages over using reinforced concrete members. The present study investigates the bending behavior of steel circular hollow sections when subjected to bending loads. The variations in material characteristics with regard to position along the cross-section of a steel tube member is first considered in this experimental study, providing for a more accurate definition of the material behavior in the model. A supported beam tested by two-point loads is the loading type that is used to study the bending performance of steel tubes. Ten circular hollow beam specimens were prepared and tested up to and post the failure stage with the following dimensions: thickness (2, 3, and 6 mm), diameter (76.2, 101.6, and 219 mm), and span (1000, 1500, and 2000 mm). A finite element analysis has been conducted for these ten specimens using the ANSYS program. The finite element model is compared to experimentally obtained data to verify that both local and global behaviors are correctly considered. The load-deflection results of this analysis showed a good agreement with the experimental results. A parametric study also was performed that considered two variables, which were the effect of the presence of circular rings and the change of opening location in the length direction on the specimens’ behavior. This study showed that the presence of the circular rings in the specimen led to an increase in its ultimate strength (of 53.24%) compared with the non-presence of these rings. In contrast, the presence of openings at 30, 40, and 50% from the specimen length reduced the strength capacity by 8.76, 14.23, and 17.88%, respectively.

## 1. Introduction

High-strength welded steel tubes utilized as structural components in buildings and other structures and a range of manufactured items are referred to as hollow structural sections [1,2,3,4]. They are manufactured to ASTM A500, A1085, and A1065 requirements in round, square, and rectangular forms and a wide range of sizes [5]. A circular hollow steel tube has been shown to be a strong structural element for constructions, jibs, skyscrapers, cranes, barriers, and fluid mechanics equipment [6,7]. The above structural system has inherent efficiency because of its ideal compression, tension, torsion, and biaxial bending properties [8]. However, the widespread usage of steel tube in earthquake applications for the construction industry is restricted because of an incomplete understanding of their behavior after being subjected to bending loads and a probable absence of flexibility and steady behavior over several cycles of loads [8,9,10]. 

Construction applications of steel tube have been confined to static loads on column members, truss components, bracing, and wall elements [11]. The advanced seismic frame uses, such as for column and beam structural elements, can significantly benefit lower seismic weight, reduced lateral bracing, modular construction applications, and innovative rehabilitation procedures [8,12]. Due to a large percentage of inelastic damage occurring in the beam elements, existing seismic standard codes require a strong-column weak-beam mechanism during their design [13,14,15]. A better understanding of the bending behavior of these elements and an appropriate method of modeling this behavior is required before steel tubes can be used for seismic design applications [16,17,18].

Until the present time, most studies on steel tube flexural behavior have been limited to beam-column structural elements. Dwyer and Galambos [19] studied various beam-column specimens to collapse, emphasizing the relevance of the ratio of axial load (P/P_y_) and the ratio of slenderness (L/r). The influence of the slenderness ratio and axial load ratio on the bending behavior was investigated by Nakashima and Liu [9] in order to obtain a better knowledge of the plastic behavior of steel tube columns in seismic constructions. To explain the plastic behavior of a steel tube column under varied axial load conditions, Wang et al. [12] conducted hybrid experimental works. For both concrete-filled tube (CFT) and hollow sections, further investigations looked at the behavior connections between broad flange beams and steel tube columns [20,21]. Several experimental studies focused on the bending behavior of steel tube beam elements under a range of monotonic loading regimes. The aspect ratio (b/h), depth-to-thickness ratio (h/t), and width-to-thickness ratio (b/t) all had a role in these experiments [22,23]. The flexural behavior of steel tube beam members under bending stresses was studied in more recent experimental work [13,14]. The findings of these experimental works emphasize the relevance of the h/t and b/t found throughout experimental testing and give insight into the predicted local buckling behavior. Lateral constraint, bending moment variation, and overall member slenderness have influenced steel tube performance under repeated bending loads [22,24]. Yet, experimental findings are confined to a tiny subset of steel tube elements, necessitating further research into their bending behavior. Numerical simulations have also been carried out to understand better the behavior of steel tube elements in structures and the various failure scenarios [25,26]. 

Sohal and Chen [27] investigated the buckling behavior of circular steel tubes. They carried out a static model study that could be used to evaluate the bending behavior depending on various hypotheses such as critical strain, buckle form and propagation, and stress in the steel tube element. To determine the post-crack flexural behavior of the steel tube column, Key et al. [28] proposed a numerical plastic method. The buckling yield line model comprises corner yielding, plate folding, and folding corner restraint components, yet the validity of these methods under bending stresses is uncertain. Similar analytical methods have adequately used FEM to simulate steel tube column and beam-column behavior. Nakashima and Liu [9] investigated steel tube columns under bending loads with varying axial load ratios until collapse using FEM. This investigation detected the buckling behavior and highlighted the significance of the axial force amount and its influence on the hysteresis loop. Goto et al. [29] used a three-surface cyclic metal plasticity material model to describe massive steel tube columns, which may offer extremely accurate findings once adjusted to experimentally obtained data. Kurata et al. [30] proposed an empirical model that predicts the slope produced by deterioration by considering the influence of the axile load ratio and slenderness ratio on the bending behavior. 

Fiber elements with constitutive characteristics for both concrete and steel were used in a recently developed model that accounts for concrete core confinement and local buckling of the steel tube [31,32,33] It has also been demonstrated that FEM can be developed to simulate the failure and ductility phase of wide-flange beam elements with global and local instabilities, resulting in a reliable approach for evaluating the beam’s ductility depending on the geometry of the cross-section, yield strength, unbraced length, strain hardening behavior, and yield ratio [34]. Square and rectangular steel tube beam section models are more constrained, mainly focusing on sections subjected to monotonic bending stresses [35]. This model used defects in the section geometry to simulate buckling and load-displacement in experimental testing. The results of this model confirm the relevance of the b/t and h/t ratios in determining local buckling behavior. Still, it has yet to be demonstrated that such an approach is relevant to members subjected to higher bending loads.

A detailed FEM study was carried out to further investigate the behavior of steel tube members under bending. This investigation contributes to the limited experimental data on the bending behavior of steel tube elements under significant bending loads by specifying limiting parameters for their usage in bending applications. The change in material characteristics with regard to position along the cross-section of a steel tube element was first considered in this experimental investigation, providing more knowledge about the material behavior. After that, the FEM model was compared with experimentally obtained data to verify that both global and local behaviors were appropriately modeled. A parametric investigation of six different steel tube characteristics (position of the opening and presence of rings in the length of steel tube) was carried out using this model, providing essential information on ductility, ultimate deflection, and ultimate load with bending.

It has been observed through the literature that there are many existing finite element analyses to predict the bending behaviour of steel tubes [36,37,38,39]. However, there are no available finite element analyses to evaluate and predict the bending behaviour of circular hollow steel tube sections with openings and presence of rings in the length of the tube, which are necessary to investigate steel tube sections under different loadings and can be used to be a reference for future studies and provide input data for the steel tubes in the finite element (FE) models.

## 2. Experimental Study

### 2.1. Materials Properties

Steel was the only material used during the experiment. The tensile specimens were prepared according to ASTM (A370) specifications [40], and tested using the tensile test machine to determine the specimens’ ultimate stress and F_u_ the yield stress F_y_, as shown in Table 1. The specimens were loaded in the longitudinal direction in steps until failure occurred.

### 2.2. Experimental Specimens

The experimental plan included testing ten beam specimens up to and post-failure stage using different circular section sizes and with diameter to thickness ratios ranging from 16.93 to 73. These specimens were distributed as follows.

#### 2.2.1. Reference Specimen

One specimen was chosen as a reference for comparing the results. This specimen had a span of 1500 mm, a diameter of 101.6 mm, and a thickness of 3 mm.

#### 2.2.2. Modified Specimens

The modified specimens were divided into four different groups as follows.

1. First Group

This group aimed to recognize the effect of changing the thickness of the circular hollow section on mechanical behavior. The primary variable of this group was the section thickness. Two specimens, whose thickness was different and span and diameter were constant, are shown in Table 2. 

The selected variable was essential since a circular hollow steel section’s buckling behavior and bending capacity depend mainly on the diameter to thickness ratio.

2. Second Group:

This group aimed to recognize the effect of the presence of openings on the investigated mechanical properties. The primary variable of this group was the presence of square openings. Three specimens, whose openings’ number and location in the specimen were different while all other parameters were constant, are shown in Table 2. 

The selected variable is essential since the functional requirements are a significant aspect of design considerations. Therefore, the provision of steel beams with openings has become acceptable in practical applications such as ventilation, heating systems, and pipelines. These specimens were as follows.

The first specimen, BT1, was the reference specimen used without openings.

The second specimen, BT4, was a specimen with one opening located at the center of the specimen.

The third specimen, BT5, was a specimen with two openings located at the two loading points of the specimen.

The fourth specimen, BT6, was a specimen with three openings located at the center and two loading points of the specimen.

The openings used in each of these specimens were identical in their shape and dimensions, which were square shapes with lengths equal to 50 mm.

3. Third Group

This group aimed to recognize the effect of changing the span of the circular hollow specimens on their investigated properties. The primary variable of this group was the span of the beam specimen. Two specimens, whose span was different while all other parameters were constant, are shown in Table 2.

4. Fourth Group

This group aimed to recognize the effect of changing the outside diameter of the circular hollow specimen on the investigated properties. The primary variable of this group was the outside diameter of the specimen (Table 2). The two specimens had an outside diameter that was different, while all other parameters were constant.

The selected variable was essential since buckling behavior and bending capacity for circular hollow sections depend mainly on the diameter to thickness ratio of the circular sections. 

### 2.3. Test Procedure 

**Figure 1:**
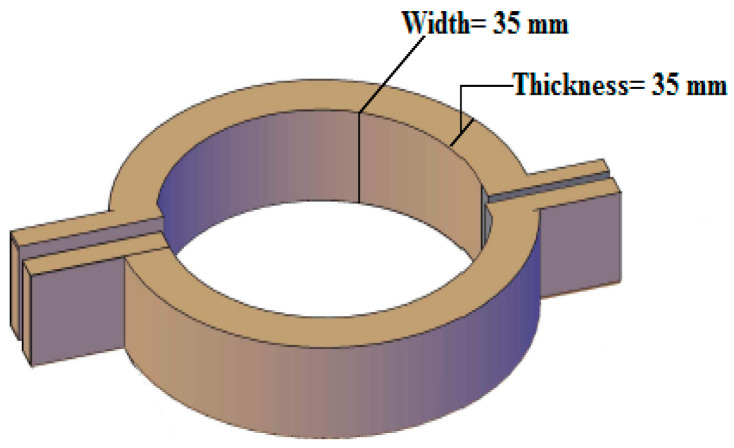
The circular rings of the experimental study.

Once hollow cylindrical tubes are bent, a deflection in their cross-section develops, resulting in ovalization. The ovalization phenomena will develop with the increase in bending, resulting in a steady decrease in the specimens’ bending stiffness. Circular hollow specimens are subjected to local buckling whenever the value of the ovalization exceeds the critical value [5]. As a result, appropriate conditions must be created at the specimen’s loading locations to avoid early ovalization and restrain the circular portions; hence the cross-section will be forced to remain circular during the loading. In this experimental work, four circular rings were employed to place the specimen at the supports and two loading positions to accomplish this. These circular rings had an internal diameter equal to the external diameter of the specimen. Each ring comprised two semi-circular pieces whose width and thickness equaled 35 mm, connected by bolts, as shown in Figure 1.

The rings acted as a stiffener for the specimens, and their functions were to:

- Ensure that the applied load was distributed axially to avoid stress concentration at a single place;
- Support the vertical load;
- Due to the high rigidity and stiffness of these rings, limit radial displacement at loading locations;
- Compress the section of the specimen so it was stiffened and supported adequately, preventing local buckling at the stress locations;
- Prevent the specimens’ sudden failure when they reached the peak load.

A four-point flexural test was employed to investigate the specimen properties. Each specimen was located between the supports. A hydraulic jack connected to a load cell and attached to the beam was used to apply a vertical load on the specimen’s center. Through the beam, the applied force was equally distributed to the specimen at two loading positions. The loading points were located at equal distances from the center of the specimen: a cylindrical roller was placed between the spreader beam and the circular rings surrounding the specimen.

### 2.4. The Measurement Instruments

#### 2.4.1. Dial Gauges

**Figure 2:**
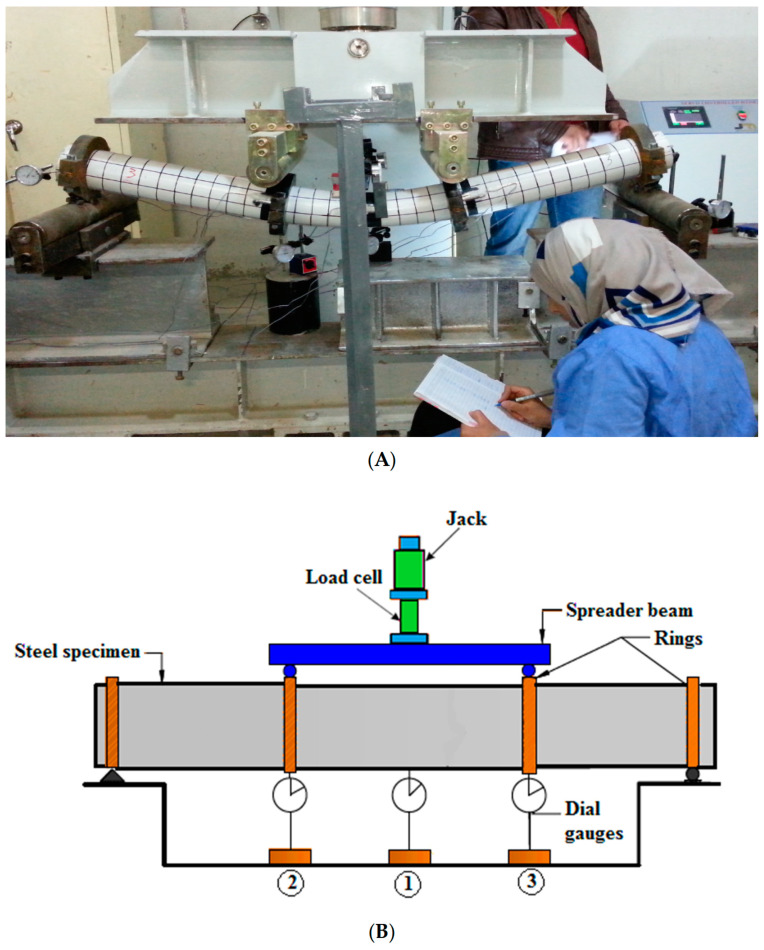
Schematic view of the test device and dial gauges’ distribution: (**A**) experimental, (**B**) numerical.

Five dial gauges were used for each specimen test and distributed as follows:

One of the dial gauges was put in the center of the specimen, and two gauges were placed at the loading points to measure the vertical deflection at these points, as shown in Figure 2.

**Figure 3:**
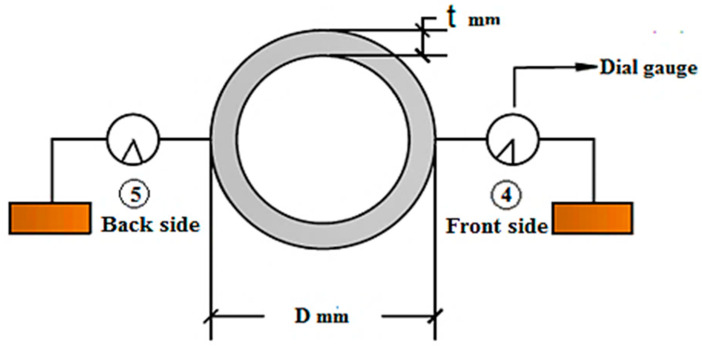
The dial gauges for measurement of the specimen ovalization.

One of the remaining dial gauges was put in the front face (front side) of the specimen and parallel to its center, while the second dial gauge was put in the back face (back side) in order to measure the ovalization deformation of the cross-section at mid-span, as shown in Figure 3.

#### 2.4.2. Data Logger 

A data logger was used to record all data measured using the strain gauges in the specimen, providing the dependence of strain with the time.

#### 2.4.3. Strain Gauges

Twelve strain gauges whose length was equal to 10 mm were used for each specimen to measure its strain, and a TML strain gauge was used in this study. The use of the mentioned strain gauges was justified by the high degree of stability, accuracy, and easy use. 

The PFL-10-11-3L strain gauge type was used in the present study with the following properties: a single element had a pre-attached vinyl lead wire with 3 m. The gauge had a length of 10 mm, a width of 2.3 mm, a gauge factor of 2.12 ± 1%, and a resistance of 119.5 ± 0.5 Ω.

**Figure 4:**
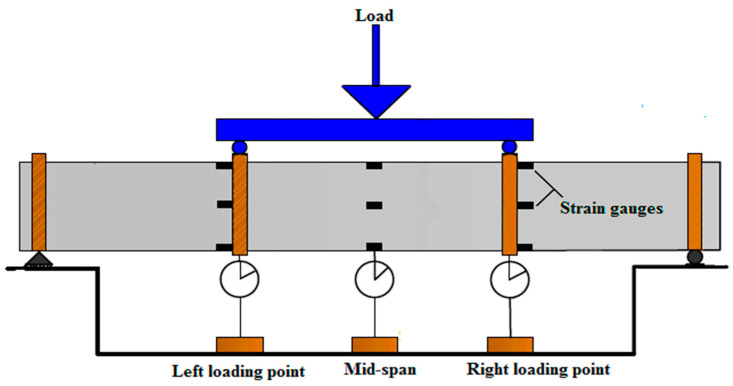
Strain gauges’ distribution on each specimen.

Four strain gauges were used in each location, as shown in Figure 4. These strain gauges were identically distributed at the center of the specimen and two loading points. The strain gauges were distributed at the top, the bottom part of the cross-section, front face, and back face to measure the specimen strain at these locations.

For installation of the strain gauges on the specimen, the following procedure was adopted.

Preparation of the surface: the bonding area was cleaned of rust, grease, and paint and uniformly finished using abrasive paper #120 to 180.

Fine cleaning: The bonding area was cleaned using a cloth soaked in acetone.

Bonding the strain gauge: the adhesive CN series was used to bond the strain gauges and cured at room temperature with the required time being approximately 40–120 s.

Coating the strain gauge: a SB tap was used to coat the strain gauges with 5–10 mm length. This offered excellent water and moisture-resistant characteristics.

## 3. Numerical Analysis (Finite Element Method (FEM))

FEM is a numerical method that is based on dividing the structures into small elements which connect to each other by their nodes. This method can be used for obtaining an approximate solution to different problems in many fields of engineering, such as linear and nonlinear problems in stress analysis, fluid flow, and heat transfer (ANSYS, 2013).

In this study, version 13 of the ANSYS program was used to develop three-dimensional finite element models and simulate the material behavior and geometrics for circular hollow specimens in order to compare the experimental results with the numerical results.

### 3.1. The ANSYS Models

#### 3.1.1. Types of Element and Material Properties

SHELL181 and SOLID45 were used in the ANSYS program for modeling all tested circular hollow specimens and the models of the parametric case studies, which were as follows.

- SHELL181

This element is suitable for analysis of shell structures that have small or moderate thickness. This element has four nodes; each node owns six degrees of freedom which are the translation in x, y, and z directions and the rotation about x, y, and z axes (ANSYS, 2013).

SHELL181 was used for modeling the hollow steel specimens as three-dimensional structural elements.

- SOLID45

SOLID45 is a suitable element for three-dimensional modeling of the solid structures. This element has eight nodes with three degrees of freedom at each node, which are the translation in x, y, and z directions (ANSYS, 2013). SOLID45 was used for modeling the steel rings as three-dimensional structural elements. 

In modeling these elements, the bilinear isotropic BISO model was used for simulating the stress–strain curve of the steel circular hollow specimens, which assumed the structural behavior as elastic–perfectly plastic. The bilinear model required the value of yield stress (F_y_, which was obtained from the material tests that were used for modeling these specimens) and the tangent modulus (ET = 10% from the modulus of elasticity).

#### 3.1.2. Modeling and Meshing of Circular Hollow Specimens

**Figure 5:**
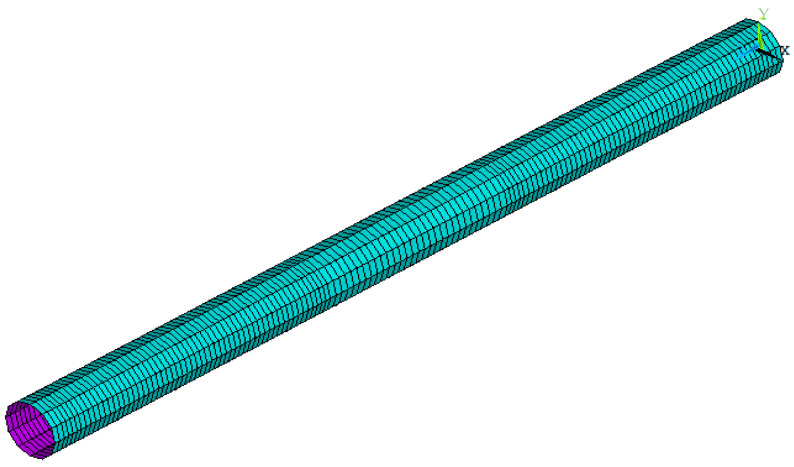
Modeling and meshing of the steel circular hollow specimens.

The SHELL181 element was used for representing the steel circular hollow specimens. The modeling of circular hollow specimens was initiated by defining the key points, drawing straight lines between these key points, and making an extrusion for the lines about the axis in order to create the areas for these specimens. The meshing of these specimens was done by dividing the longitudinal direction into many elements with lengths equal to 10 mm for each one, while the transverse direction was divided into twelve elements; thus, the number of elements became 1944 elements. Figure 5 shows the modeling and meshing of the steel circular hollow specimens.

#### 3.1.3. Modeling and Meshing of Circular Rings

**Figure 6:**
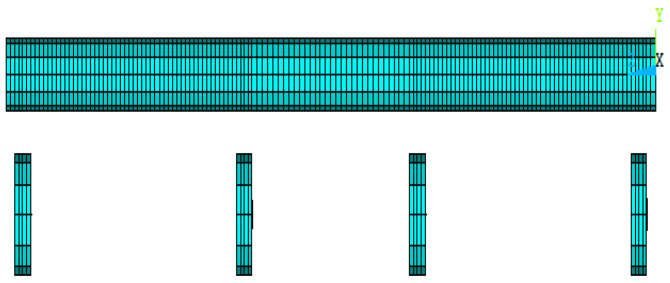
Modeling and meshing of the steel circular hollow specimens.

**Figure 7:**
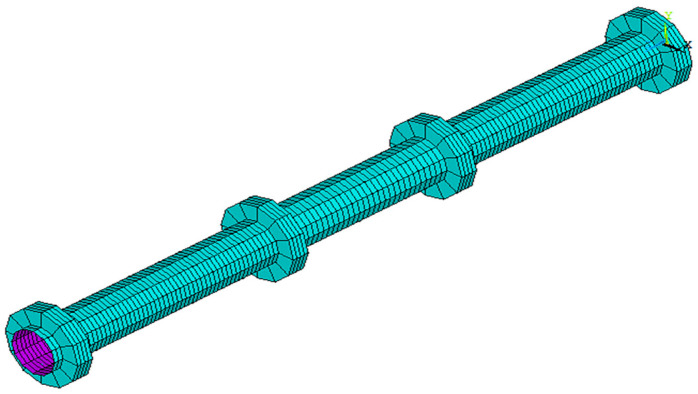
The merge of the circular hollow specimens and rings.

For representing the steel rings, we used the SOLID45 element. Modeling the steel rings started by creating annulus areas at the supports and two loading points at a distance y from the specimen center. After that, it was made to extrude for these areas in a longitudinal direction with a length equaling 35 mm in order to create circular rings. At the same time, the meshing of these rings was done by using the same mesh style as of the steel circular specimens, as shown in Figure 6. After that, the circular rings were moved by the same y distance but in the opposite direction, and a merger of all items was done in order to make one block as a circular specimen, as shown in Figure 7.

#### 3.1.4. Boundary Conditions and Applied Load

**Figure 8:**
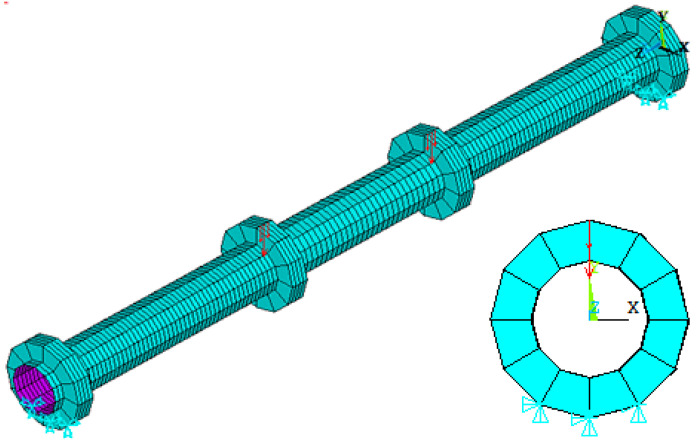
Boundary conditions and applied load on the specimen.

For constructing the models, two simple supports were modeled in the middle nodes of the rings (37.5 mm) away from the edge of the circular specimen. Both supports were a hinge (Uy = 0), (Ux = 0) and (Uz = 0). The applied load on the specimen was equally divided by the rings located at the loading points. The load at each ring was distributed to three nodes; half of this load was applied on the middle node, and the remaining half was equally divided between the terminal nodes, as shown in Figure 8.

The ANSYS Newton–Raphson approach was used to solve nonlinear problems. In this approach, the applied load is separated into a set of load increments defined as load steps. Before carrying on to the next load increment, the stiffness matrix of the model was modified at the end of each incremental solution to represent nonlinear changes in structural stiffness. To achieve convergence, a solution was produced by solving many sub-steps in each load step. Before carrying on to the next sub-step, a repetition was carried out of each sub-step until a convergent solution was obtained.

## 4. Comparison between the Experimental and Numerical Results

**Figure 9:**
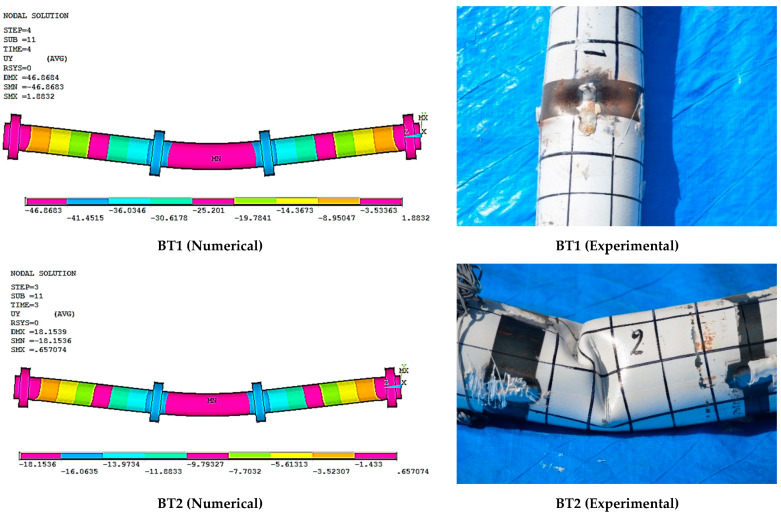

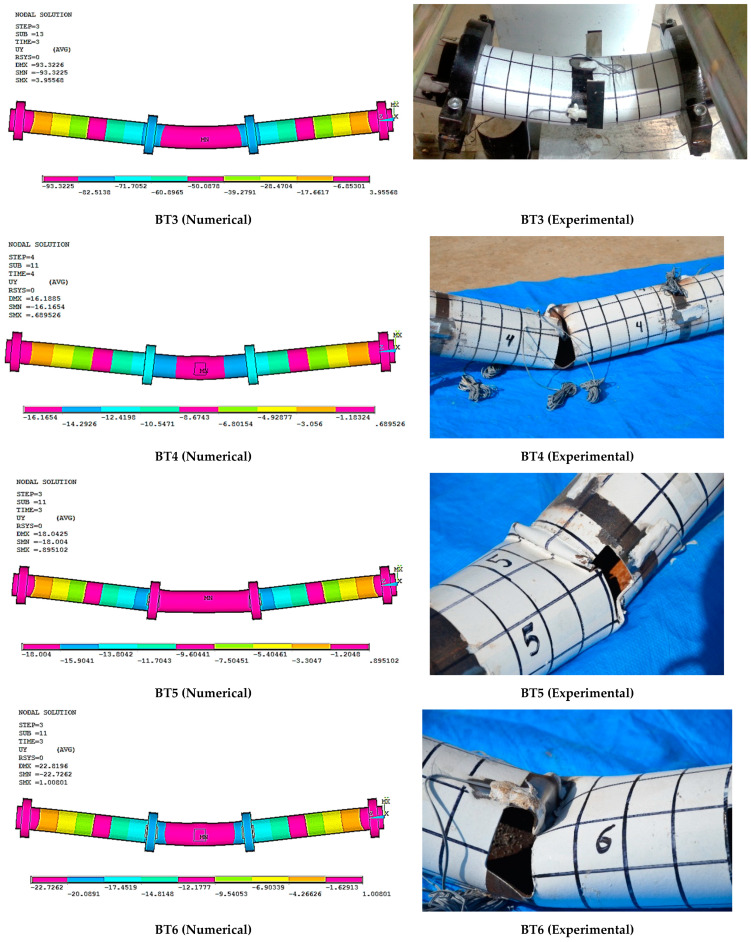

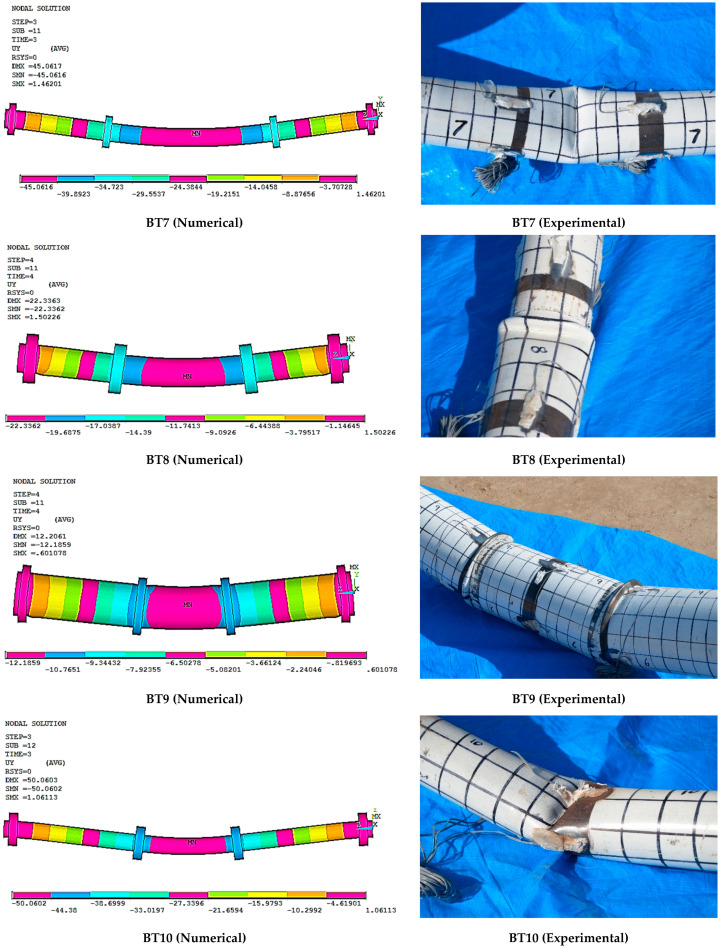
The displacement of specimens BT1 to BT10.

This part presents the comparison of load-deflection curves between the models of the finite element with the experimental results of steel circular hollow specimens. Therefore, it was necessary to plot the vertical loads against the deflection at the specimen mid-span in order to verify this method. These specimens showed a good agreement between the numerical method and the experimental results, as shown in Table 3. However, there were slight differences between experimental and FEM results. That might be attributed to the friction forces which appear at the supports and loading rollers while performing the four-point test, but it is hard to measure such forces under real test conditions. The friction forces are, therefore, simulated approximately under real conditions. Slipping also occurs; thus, this assumption would not be correct. These variables influenced the outcomes and are the major reasons why the simulated and experimental outcomes were inconsistent. The numerical displacement for specimens BT1 to BT10 are shown in Figure 9.

## 5. Parametric Case Studies

Case studies carried out in this research were divided into two groups as follows.

- First group

**Figure 10:**
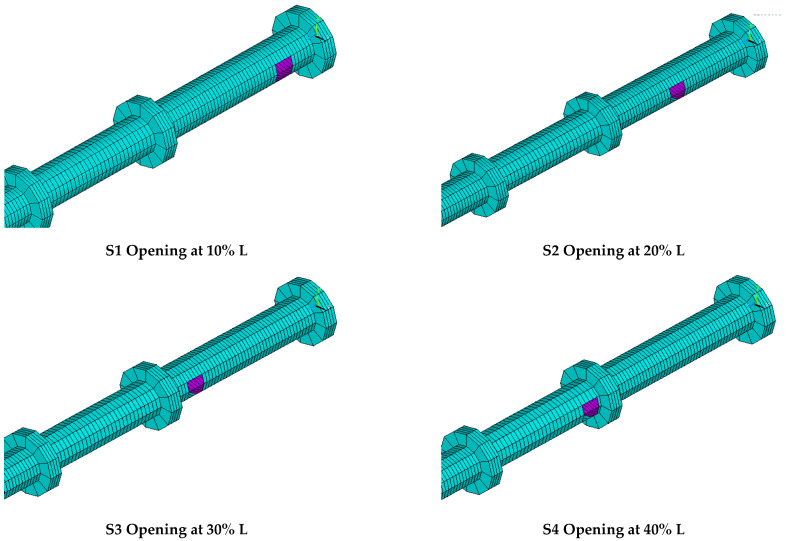

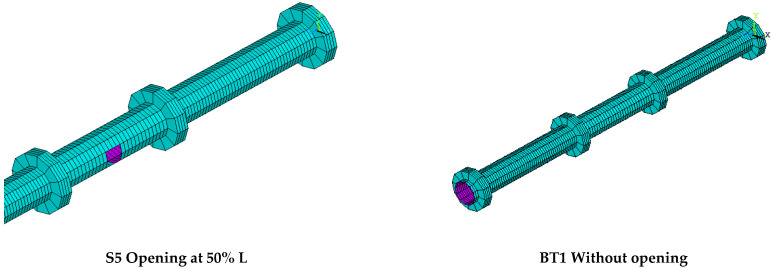
The first group of specimens of parametric case studies.

This group included the study of five circular hollow specimens S1, S2, S3, S4, and S5, as shown in Table 4, in addition to the reference specimen BT1, in order to compare the results of these specimens. These specimens had the same diameter, thickness, and span with the presence of one opening in each specimen; the primary variable in this group was the location of the opening in specimen length, as shown in Figure 10. The reason for studying this variable was in order to study the effect of opening location on the structural behavior, strength, and failure of these specimens.

- Second group

**Figure 11:**
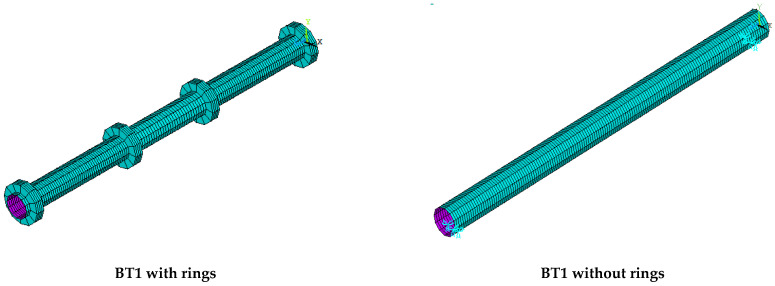
The second group of specimens of parametric case studies.

The second group of the parametric case studies included the study of the effect of the presence of rings on the structural behavior using one specimen, S6, and comparing it with the reference specimen BT1, as shown in Table 5. These specimens had the same diameter, thickness and span; the primary variable in this group was the presence or absence of the circular rings, as shown in Figure 11.

## 6. Results and Discussion

### 6.1. First Group

**Figure 12:**
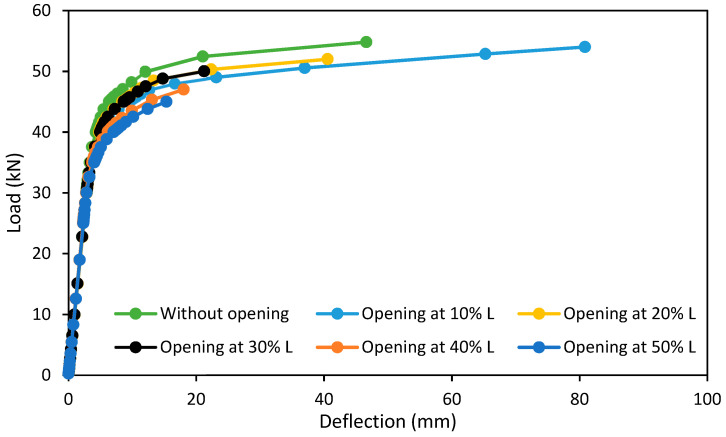
Load-deflection curve of the first group specimens in parametric case studies.

Figure 12 gives the load-deflection curve of the first group of specimens used for parametric case studies, which had different opening locations along the specimen. From this figure, it can be noted that specimens S1, S2, S3, S4, and S5 behaved in a similar way to the reference specimen BT1. This behavior is represented in three basic stages, which are:

- The linear elastic stage: in this stage, the specimen exhibited a linear relationship between the vertical load and the resulting deflection. This stage is represented by an inclined line with a high inclination as a result of a high increase in the applied loads compared with a little increase in the specimen deflection.
- The ovalization stage: this stage is represented by a flat line with a small slope as a result of a high increase in the specimen deflection compared with a little increase in the specimen loads.
- The failure stage: this stage begins at ultimate load and refers to the specimen’s structural collapse.

From this, it can be noted that the change of opening location in the specimens did not affect the general behavior of these specimens but affected the specimens’ strength capacity and their deflection values, and this led to different locations of failure.

The presence of an opening at 10% L of the specimen S1 increased its deflection at the ultimate load by 76.64% compared with the reference specimen BT1 as a result of the presence of an opening near the support, which led to the weakness of this region. The high response of this specimen caused an increase in its ductility by 47.84% compared with that of the reference specimen BT1. At the same time, the differences in strength capacity at the ultimate point were few and did not exceed 2% compared with BT1; this is because the location of the opening was far from the critical region (pure bending region). 

**Figure 13:**
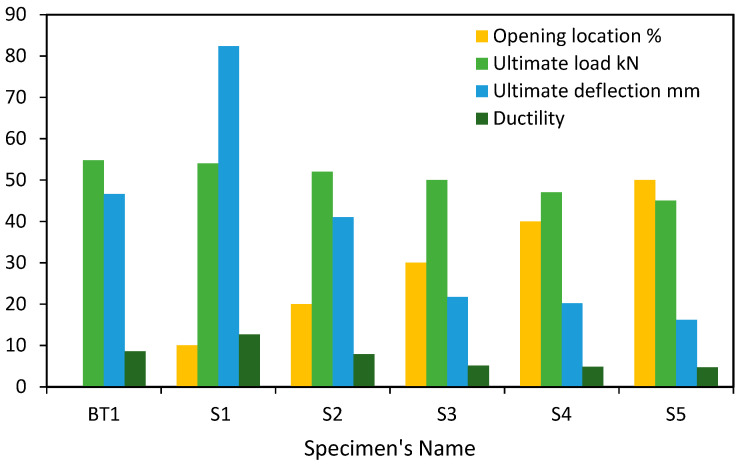
Results of the first group specimens in parametric case studies.

The presence of an opening at 20% L of specimen S2 reduced the strength capacity and the deflection value at the ultimate point by 5.11 and 12.05%, respectively, compared with that of BT1, as shown in Figure 13.

For specimens S3, S4, and S5, the presence of openings at 30, 40, and 50% from the specimen length reduced the strength capacity by 8.76, 14.23, and 17.88%, respectively, compared with that of BT1.

From this figure, it can be noted that the deflections of specimens S3, S4, and S5 at the ultimate load were few compared with the reference specimen BT1. From this, we note that the presence of an opening in the critical region (at the loading points and pure bending region) reduced the structural strength capacity and the ultimate deflection of the specimens, which led to reduced resistance to collapse.

### 6.2. Second Group 

**Figure 14:**
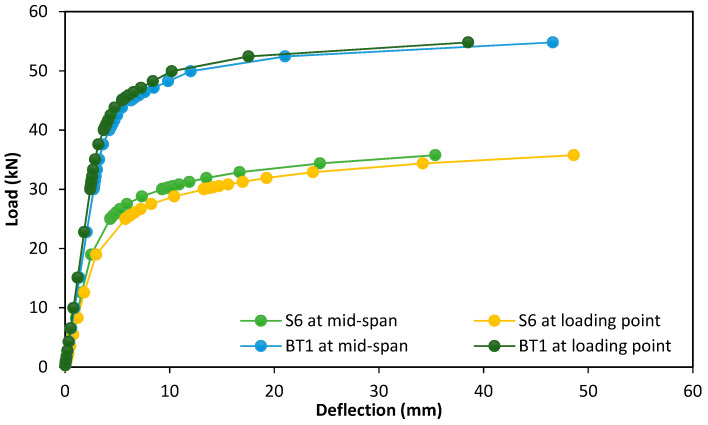
Load-deflection curve of the second group specimens in parametric case studies.

When comparing the second group specimens of parametric case studies BT1 and S6 with and without rings, as shown in Figure 14, it was observed that the maximum deflections at the ultimate load for these specimens were close to each other, and the percentage of difference did not exceed 5%, but they were different in their location of maximum deflection and failure mode.

**Figure 15:**
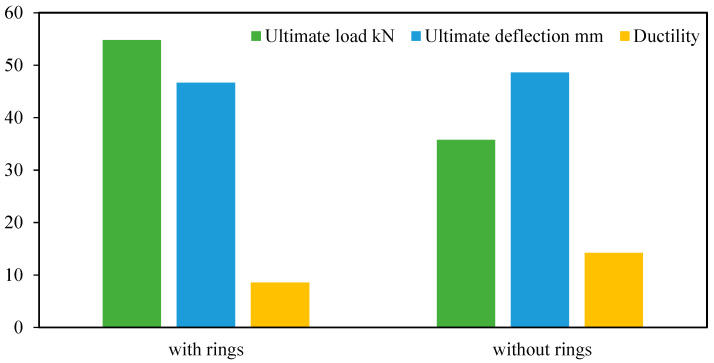
Results of the second group specimens in parametric case studies.

From the beginning of loading to the ultimate load, specimen S6 showed greater deflection at the loading points. At the ultimate load, it was observed that this deflection increased by 37.42% from the mid-span deflection and thus led to the local buckling formation at these points, unlike the reference specimen BT1, which gave its maximum deflection at the mid-span. The presence of rings of the reference specimen BT1 at loading points worked to increase the specimen strength capacity by 53.24% compared with that of the specimen S6, as shown in Figure 15. From this figure, it can be noted that the presence of rings at loading points enhanced and restricted the loading points and reduced their effect on applied loads, thus preventing the local buckling failure at these points; at the same time, these rings increased the specimens’ load capacity.

**Figure 16:**
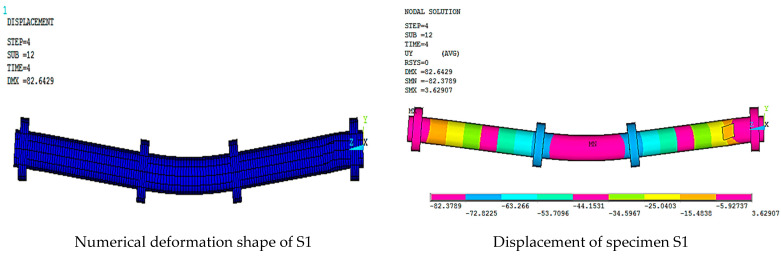

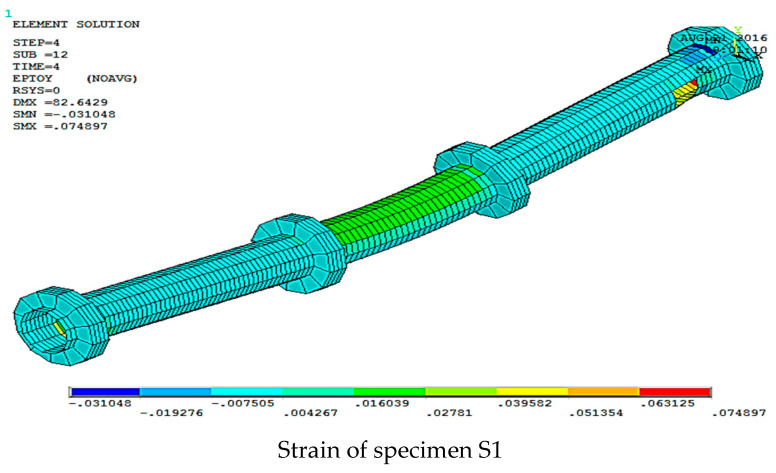
Numerical analysis of S1.

**Figure 17:**
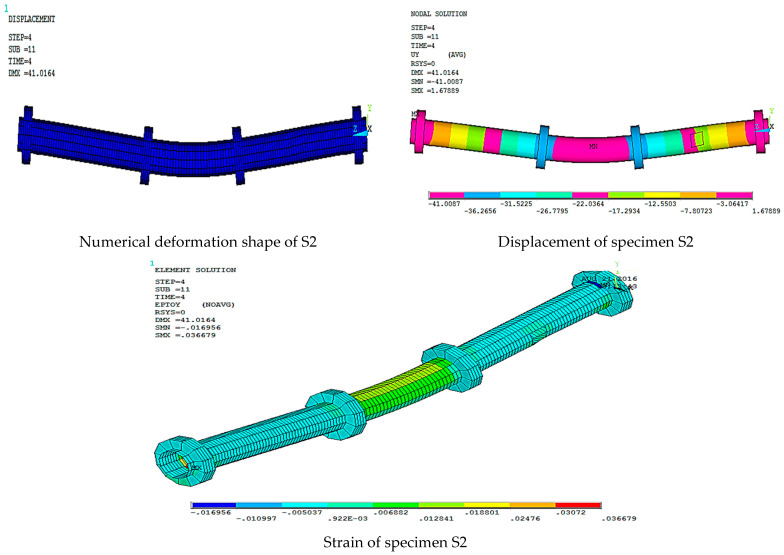
Numerical analysis of S2.

**Figure 18:**
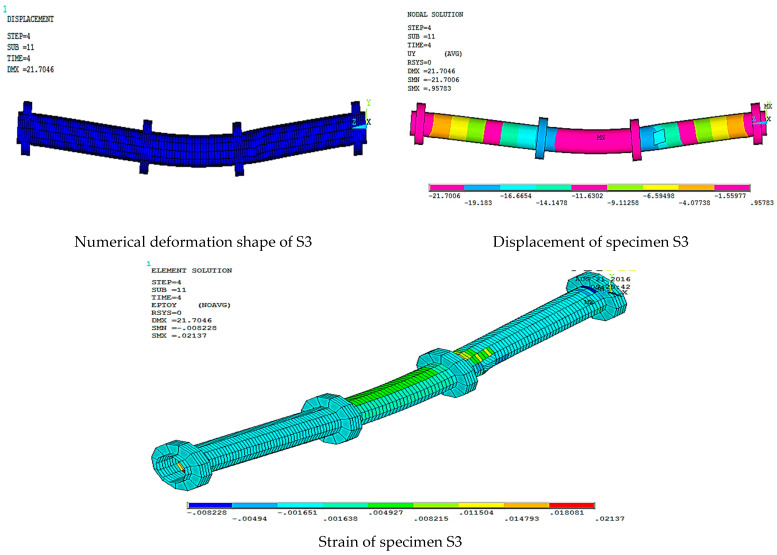
Numerical analysis of S3.

**Figure 19:**
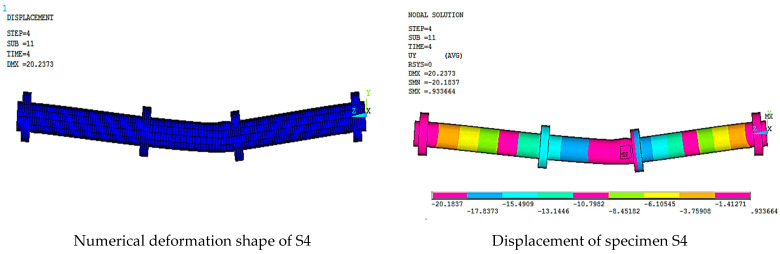

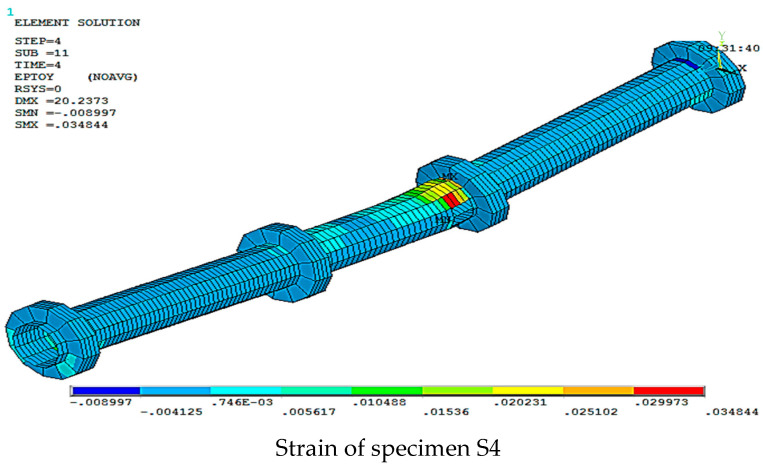
Numerical analysis of S4.

**Figure 20:**
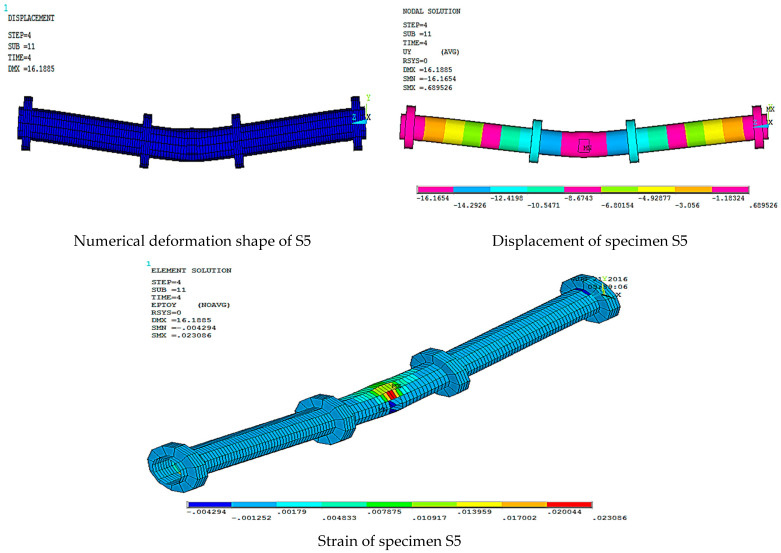
Numerical analysis of S5.

**Figure 21:**
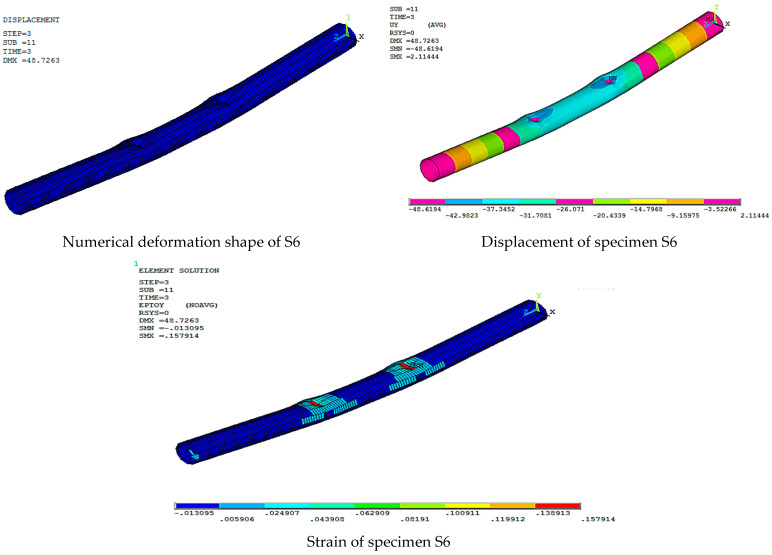
Numerical analysis of S6.

Figure 16, Figure 17, Figure 18, Figure 19, Figure 20 and Figure 21 give the displacement, deformed shape, and strain of all specimens of all parametric case studies. From these figures, it can be noted that the numerical displacement of the specimens S1, S2, S3, S4, and S5 changed with changing the location of opening along the specimen length.

Specimens S1 and S2 showed a high displacement when the opening location was at 10 and 20% from the specimen length and gave maximum displacement at the specimen mid-span, equal to 82.37 and 41.01 mm, respectively. However, when the opening location approached from the pure bending region, we observed that the displacement for the specimens S3, S4, and S5 was reduced and gave maximum displacement equal to 21.70, 20.18, and 16.16 mm, and this indicated that the distance of the opening location to the pure bending region made the specimens less resistant to failure.

While specimen S6 gave maximum displacement at two loading points equal to 48.62 mm, the absence of the circular rings made the specimen less resistant to failure and led to the occurrence of local buckling failure at the two loading points.

## 7. Conclusions

In this experimental investigation, a numerical model of steel tube sections under flexure was carried out after the comparison and validation of a FEM model during serious experimental testing. The experimental investigation considered six different sections with two variables, which were the effect of the presence of circular rings and the change of opening location in the length direction on the specimens’ behavior. All steel tube sections were tested under the same loading protocols simulating the effect of bending load. The key conclusions associated with this work are summarized as follows:

- The load-deflection results of this analysis showed a good agreement with the experimental results.
- The change of opening location in the specimens did not affect the general behavior of these specimens but affected the specimens’ strength capacity and their deflection values, and this led to different locations of failure.
- The presence of an opening in the critical region (at the loading points and pure bending region) reduced the structural strength capacity and the ultimate deflection of the specimens, which led to reduced resistance to collapse.
- The presence of rings at loading points enhanced and restricted the loading points and reduced their effect on applied loads, thus preventing the local buckling failure at these points; at the same time, these rings increased the specimens’ load capacity.

## Figures and Tables

**Table 1:** Tensile test results of the specimens.

Label	Thickness t (cm)	Ultimate Stress F_u_ (MPa)	Yield Stress F_y_ (MPa)
BT1	0.3	370	290
BT2	0.2	430	360
BT3	0.6	440	285
BT4	0.3	370	290
BT5	0.3	370	290
BT6	0.3	370	290
BT7	0.3	370	290
BT8	0.3	370	290
BT9	0.3	370	290
BT10	0.3	370	290

**Table 2:** Test group specimens.

Label	Diameter D (mm)	Span L (mm)	Thickness t (mm)	D/t
BT1	101.6	1500	3	33.87
BT2	101.6	1500	2	50.80
BT3	101.6	1500	6	16.93
BT4	101.6	1500	3	33.87
BT5	101.6	1500	3	33.87
BT6	101.6	1500	3	33.87
BT7	101.6	2000	3	33.87
BT8	101.6	1000	3	33.87
BT9	219.0	1500	3	73.00
BT10	76.20	1500	3	25.40

**Table 3:** Comparison between the experimental and numerical results.

Label	Exp. Deflection at Ultimate Load (mm)	FEM Deflection at Ultimate Load (P_u_) (mm)	Deflection FEM/Exp. (%)	Absolute Percentage Error
**BT1**	51.88	46. 86	90.33	9.67
**BT2**	22.10	18.15	82.13	17.8733
**BT3**	108.06	93.32	86.36	13.64057
**BT4**	17.57	16.16	91.97	8.025043
**BT5**	20.48	18.00	87.89	12.10938
**BT6**	24.35	22.72	93.31	6.694045
**BT7**	55.31	45.06	81.47	18.53191
**BT8**	25.80	22.33	86.55	13.44961
**BT9**	14.15	12.18	86.08	13.92226
**BT10**	58.25	50.06	85.94	14.06009

**Table 4:** The first group of specimens of parametric case studies.

Label	t (mm)	D (mm)	L (mm)	Primary Variable
BT1	3	101.6	1500	Without opening
S1	3	101.6	1500	Opening at 10% L
S2	3	101.6	1500	Opening at 20% L
S3	3	101.6	1500	Opening at 30% L
S4	3	101.6	1500	Opening at 40% L
S5	3	101.6	1500	Opening at 50% L

**Table 5:** The second group of specimens of parametric case studies.

Label	t (mm)	D (mm)	L (mm)	Primary Variable
BT1	3	101.6	1500	With rings
S6	3	101.6	1500	Without rings

## Data Availability

The data used to support the findings of this study are included within the article.
